# Bone Marrow Protein Oxidation in Response to Ionizing Radiation in C57BL/6J Mice

**DOI:** 10.3390/proteomes2030291

**Published:** 2014-06-25

**Authors:** Yong-Chul Kim, Michal Barshishat-Kupper, Elizabeth A. McCart, Gregory P. Mueller, Regina M. Day

**Affiliations:** 1 Department of Pharmacology, Uniformed Services University of the Health Sciences, Bethesda, MD 20814, USA; E-Mails: yongchul.kim.ctr@usuhs.edu (Y.-C.K.); michalkupper@gmail.com (M.B.-K.); elizabeth.mccart@usuhs.edu (E.A.M.); 2 Department of Anatomy, Physiology and Genetics, Uniformed Services University of the Health Sciences, Bethesda, MD 20814, USA; E-Mail: gregory.mueller@usuhs.edu

**Keywords:** acute radiation syndrome, protein carbonylation, proteomic analysis

## Abstract

The bone marrow is one of the most radio-sensitive tissues. Accidental ionizing radiation exposure can damage mature blood cells and hematopoietic progenitor/stem cells, and mortality can result from hematopoietic insufficiency and infection. Ionizing radiation induces alterations in gene and protein expression in hematopoietic tissue. Here we investigated radiation effects on protein carbonylation, a primary marker for protein oxidative damage. C57BL/6 mice were either sham irradiated or exposed to 7.5 Gy ^60^Co (0.6 Gy/min) total body irradiation. Bone marrow was obtained 24 h post-irradiation. Two dimensional (2-D) gel electrophoresis and Oxyblot immunodetection were used to discover carbonylated proteins, and peptide mass fingerprinting was performed for identification. 2D gels allowed the detection of 13 carbonylated proteins in the bone marrow; seven of these were identified, with two pairs of the same protein. Baseline levels of carbonylation were found in 78 kDa glucose-related protein, heat shock protein cognate 71 KDa, actin, chitinase-like protein 3 (CHI3L1), and carbonic anhydrase 2 (CAII). Radiation increased carbonylation in four proteins, including CHI3L1 and CAII, and induced carbonylation of one additional protein (not identified). Our findings indicate that the profile of specific protein carbonylation in bone marrow is substantially altered by ionizing radiation. Accordingly, protein oxidation may be a mechanism for reduced cell viability.

## 1. Introduction

High dose total body irradiation may occur as the result of a nuclear accident or terrorist event. Radiotherapy is also a common therapeutic modality for cancer treatment, including for leukemia and lymphoma. Unfortunately, radiation therapy is not tumor-specific and underlying normal tissues, particularly the bone marrow, are extremely vulnerable to radiation injury. The acute hematopoietic effects of total body exposure to ionizing radiation in humans, termed Hematopoietic Syndrome, usually occur within ~30–60 days following exposures greater than 2 Gy. In the extreme, this condition results in death from hematopoietic insufficiency and infection [1]. 

Ionizing radiation destroys both mature blood cells and hematopoietic progenitor/stem cells in the bone marrow compartment, reducing the number of resident, tissue-specific adult stem cells that are critical for hematopoietic repair and regeneration. While loss of mature blood cells is a key factor in radiation morbidity, mortality is believed to occur due to prolonged myelosuppression from the loss of hematopoietic progenitor cells (HPC) and primitive hematopoietic stem cells (HSC) [2,3]. A delicate balance between HSC self-renewal, proliferation, and differentiation is required to ensure proper bone marrow HSC repopulation, progenitor cell reconstitution, and mature blood cell production [4]. Normally, HSC quiescence ensures life-long maintenance of the HSC pool, protecting against premature exhaustion of hematopoietic potential [5].

Studies have shown that high dose ionizing radiation induces senescence and apoptosis of the HSC resulting in exhaustion of the stem cell pool [6,7,8]. Work from our laboratory demonstrated that Lin^-^ bone marrow progenitor cells from mice exposed to 7.5 Gy total body irradiation exhibited decreased expression of genes associated with cellular proliferation and progression of the cell cycle and increased expression of genes associated with cell cycle checkpoints and cell cycle arrest [6]. The expression of key cell cycle regulation and checkpoint genes Ak1, Ccna1, Cdkn1a, Cdkn1b, Cdkn2a, Hus1, Npm2, Prmp1, Pmp22 and Trp63 were increased in response to total body irradiation [6]. Subsequent microarray analyses from other investigators indicated that up to 1302 genes were differentially expressed between 3–21 days postirradiation, affecting multiple biological processes including self-renewal, hematopoiesis, adhesion, differentiation, and immune response [8,9,10]. Additionally, ATM/CHEK2/p53 and AKT/PI3K signaling pathways were shown to be activated for as long as 4 weeks postirradiation, correlating with prolonged pro-apoptotic signaling following radiation exposure [8,9]. Experiments using qRT-PCR directed toward the detection of specific gene products associated with programmed cell death demonstrated increased expression of Bax and caspase-9 in total bone marrow cells following radiation exposure [11]. Thus, altered gene expression correlates with the loss of clonogenicity of the bone marrow cells following radiation exposure due to either apoptosis and/or accelerated senescence.

A recent proteomic study compared altered protein expression in the bone marrow of CBA/Ca and C57BL/6 strains of mice following 4 Gy radiation exposure [12]. Interestingly, CBA/Ca mice are more resistant to radiation-induced hematopoietic cell damage than C57BL/6 mice, and the protein expression patterns were shown to also differ between the two strains of mice [12]. In CBA/Ca mice, 18 proteins had altered expression following radiation exposure, but in C57BL/6 mice 27 proteins were altered after radiation [12]. In both strains of mice, radiation induced the expression of serum albumin, apolipoprotein A-1, α-ferroprotein, haptoglobin, and α-1-antitrypsin. The proteins serotransferrin, neutrophil collagenase, and peroxiredoxin 2 were altered only in C57BL/6 mice [12]. The classifications of several of these proteins were proposed to be associated with long-term pathological impact of radiation on the bone marrow. For instance, apolipoprotein A-1, α-1-antitrypsin, and α-ferroprotein were classified as potential markers for acute-phase radiation response, potentially due to altered cytokine regulation following ionizing radiation exposure [12]. Additionally, changes in levels of neutrophil collagenase and α-1-antitrypsin were proposed to be related to increased inflammatory responses following radiation exposure [12]. Importantly, the proteomic analysis of the bone marrow tissue following radiation exposure provided a different data set of radiation-induced cellular changes compared with the data set provided by gene expression analyses, providing an increased understanding of the cellular and biological impact of radiation on the bone marrow environment.

Studies have indicated that total body irradiation causes persistent oxidative stress in the hematopoietic compartment [13,14]. Although DNA damage has long been considered the most critical biological effect of ionizing radiation [13], a number of studies have indicated that radiation damage to other biological molecules, including proteins, also has significant impact on cellular viability and clonogenicity [15,16,17]. Using primary endothelial cell cultures, we previously demonstrated that protein oxidation by radiation can lead to endoplasmic reticulum (ER) stress, that can lead to programmed cell death [17]. Here we have investigated the alterations in protein oxidation induced by radiation in the bone marrow using two dimensional (2-D) gel electrophoresis and Oxyblot detection of protein carbonylation. Peptide mass fingerprinting was then used for the identification of carbonylated proteins. The present findings show that radiation exposure alters the profile of protein carbonylation in bone marrow to produce a biosignature for radiation injury. We propose that the identification of the proteins involved will increase our overall understanding of the biological effects of ionizing radiation.

## 2. Experimental

### 2.1. Chemicals

Except where noted, chemicals were purchased from Sigma-Aldrich (St. Louis, MO, USA). 

### 2.2. Animals

C57BL/6J strain female mice (The Jackson Laboratory, Bar Harbor, ME, USA) weighing 17.5–21.5 g were 12–14 weeks of age at the time of irradiation. Mice were kept in a barrier facility for animals accredited by the Association for Assessment and Accreditation of Laboratory Animal Care International. Mice were housed in groups of four. Animal rooms were maintained at 21 ± 2 °C, 50% ± 10% humidity, and 12-h light/dark cycle with commercial freely available rodent ration (Harlan Teklad Rodent Diet 8604, Frederick, MD, USA). Acidified water (pH = 2.5–3.0) was available ad libitum to control opportunistic infections [18]. All animal handling procedures were performed in compliance with guidelines from the National Research Council for the ethical handling of laboratory animals, and were approved by the Institutional Animal Care and Use Committee of the Armed Forces Radiobiology Research Institute (AFRRI, Bethesda, MD, USA).

### 2.2. Irradiation

Mice were exposed to a whole body gamma radiation in a bilateral field at AFRRI’s ^60^Co facility. The midline tissue dose to the animals was 7.00–7.50 Gy at a dose rate of 0.6 Gy/min. Control animals were sham irradiated. The alanine/electron spin resonance (ESR) dosimetry system (American Society for Testing and Materials, Standard E 1607) was used to measure dose rates (to water) in the cores of acrylic mouse phantoms. To simulate a mouse, the phantoms were 3 inches in length and 1 inch in diameter. For field mapping, all exposure rack compartments contained phantoms, and alternate phantoms contained alanine dosimeters. The ESR signals were measured using a calibration curve based on the standard calibration dosimeters (National Institute of Standards and Technology (NIST), Gaithersburg, MD, USA). The overall uncertainty in the doses given to the calibration dosimeters at NIST was approximately 1.8% at 2 standard deviations. The accuracy of the calibration curve was verified by parallel measurements of doses to selected dosimeters at AFRRI and the National Physical Laboratory (Middlesex, UK). Corrections were applied to the dose rates in phantoms for the decay of ^60^Co and differences in the mass energy-absorption coefficients for water and soft tissue.

### 2.4. Protein Oxidation Detection by OxyBlot

Total bone marrow was obtained by flushing the femurs and tibias with phosphate buffered saline (PBS) using a 23 g needle. Bone marrow proteins were extracted using 1 mL of the following protein lysis buffer (PLYB): 10 mM Na_4_P_2_O_7_, 50 mM NaF, 50 mM NaCl,1 mM EDTA (pH = 8.0), 50 mM HEPES, 1% Triton X100, adjusted to pH = 7.5, 2 mM Na_3_VO_4_, 1 mM PMSF, 1 mM DTPA and 1 tablet/10 mL buffer of complete mini (Roche Applied Science, Indianapolis, IN, USA) with 1 tablet per 10 mL buffer of complete protease inhibitors (Roche Applied Science, Indianapolis, IN, USA). The OxyBlot oxidized protein detection kit was purchased from Chemicon (Millipore, Billerica, MA, USA). 2,4-Dinitrophenylhydrazine (DNPH) derivatization was performed for 15 min at room temperature following manufacturer’s instruction using 10 µg of protein. After DNPH derivatization, proteins were subjected to two-dimensional gel electrophoresis (see below). For immunodetection, proteins were transferred to polyvinylidene difluoride (PVDF) membranes. Membranes were blocked with 1% BSA in phosphate buffer saline (PBS) containing (w/v) 0.05% Tween-20 for 1 h at RT. After overnight 4 °C incubation with anti-DNP antibody, blots were washed three times and secondary rabbit antibody was added for 1 h at ambient temperature. Blots were washed and developed using a SuperSignal West Pico chemiluminescence detection system (Thermo Scientific, Rockford, IL, USA). As a negative control, samples were not derivatized with DNP [19]. Densitometry on scanned films used ImageJ software [20]. 

### 2.5. 2-D Gel Electrophoresis

Protein extraction from the bone marrow was performed as described for one-dimensional gels [21]. Four volumes of 10 mM DNPH (in 2 M HCl) were added to 200 μg protein extract of each sample and incubated for 30 min at room temperature. A final concentration of 15% of ice cold trichloroacetic acid (TCA) was added to each sample and incubated for 10 min on ice. Samples were centrifuged for 10 min at 16,000× *g* at 4 °C. Pellets were washed three times with ethanol ethyl acetate (1:1) and centrifuged at 16,000× *g* for 15 min, 4 °C. 2-D gel electrophoresis was performed according to manufacturer’s instructions (2-D Starter Kit, Bio-Rad Laboratories, Hercules, CA, USA). Pellets were resuspended in 2-D rehydration buffer. The first dimension separation was performed with the Protean Isoelectric Focusing (IEF) Cell (Bio-Rad Laboratories). Samples were applied to immobilized pH gradient strips (nonlinear pH 5–8) for 1 h at room temperature and then covered with mineral oil and subjected to IEF. Protein IEF strips were reduced and alkylated by incubating for 10 min each in Equil Buffers 1 and 2 according to the manufacturer’s instructions. The strips were embedded in 0.7% agarose on top of 4%–20% acrylamide gels (Criterion precast gels, Bio-Rad Laboratories) and subjected to second dimension electrophoresis. Proteins were transferred to PVDF membranes using a shortened protocol (20 min, 20 V) so that proteins remaining in the partially transferred gels could be visualized by Coomassie staining (SimplyBlue Safe Stain, Invitrogen, Carlsbad, CA, USA). Carbonylated proteins detected on the Oxyblot immunoblots were mapped to corresponding features on Commassie stained gels (Bio-Rad). The features were excised for peptide mass finger printing.

### 2.6. Peptide Mass Fingerprinting for Protein Identification

Protein identifications were assigned on the basis of peptide mass fingerprinting performed as described previously [22]. Briefly, protein spots were destained, the gel fragments were then equilibrated with 0.2 mL of 100 mM NH_4_HCO_3_/50% acetonitrile for 45 min at 37 °C, dehydrated in 100 μL 100% acetonitrile and dried under vacuum. The dried gel pieces were rehydrated with 40 mM NH_4_HCO_3_/10% acetonitrile containing 20 ng/μL trypsin (Trypsin Gold, Mass Spectrometry Grade, Promega, Madison, WI, USA) and incubated overnight at 37 °C. Peptide fragments were recovered in sequential (60 min, room temperature) extractions with 1.0% trifluoroacetic acid (TFA, 75 µL) followed by two rinses with 50% acetonitrile/5% TFA (50 µL each). The three collections were pooled, dried under vacuum and dissolved in 10 µL of 1% TFA. The peptides were then purified and concentrated using a C18 Zip Tip® (Millipore Corporation, Billerica, MA, USA) and mixed with alpha-cyanohydroxycinnamic acid matrix (10 mg/mL in 50% acetonitrile/0.1% TFA) containing bradykinin (1060.5692 daltons; 50 fmol/mL) and adrenocorticotropic hormone fragment 18–39 (2465.1989 daltons; 150 fmol/mL; AnaSpec, San Jose, CA, USA) as internal standards. Samples were analyzed by matrix-assisted laser desorption ionization time-of-flight (MALDI-TOF) mass spectrometry using a Voyager MALDI-TOF DE-STR instrument (PE Biosystems, Framingham, MA, USA). The mass spectrometer was operated in reflectron mode with an accelerating voltage of 20,000 V, a grid voltage of 76.13% and a guidewire voltage of 0.003%. Peptide mass data were used to query the National Center for Biotechnology Information (NCBI; Bethesda, MD, USA) protein sequence database accessed through the ProteinProspector MS-Fit search engine [23,24]. Protein assignments were made on two criteria: (1) probability scores derived from the Molecular Weight Search (MOWSE) of ProteinProsector, based upon mass matches and percent protein sequence coverage, and (2) minimal frequency of three observations across four separate experiments. Published evidence supporting the assignments was also taken into account.

## 3. Results and Discussion

### 3.1. Radiation-Induced Protein Carbonylation in Bone Marrow Tissue

**Figure 1:**
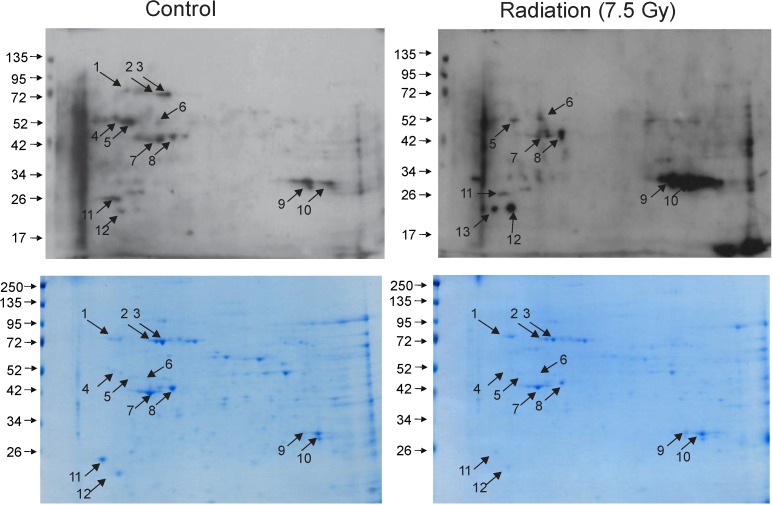
Two dimensional gel electrophoresis and Oxtblot analysis of sham-irradiated and irradiated bone marrow proteins. C57BL/6 mice were either sham irradiated (control) or exposed to 7.5 Gy TBI. Bone marrow cell lysates were obtained 24 h postirradiation, and protein lysates were combined from 3 mice for one gel. Upper panels: Oxyblot. Lower panels: Coomassie stained gels. Proteins detected by Oxyblot are indicated with numbered arrows. Immunoblots and gels are representative.

The present investigation revealed that ionizing radiation substantially alters the pattern of protein carbonylation in bone marrow, suggesting a causative mechanism for radiation-induced disruptions in hematopoiesis. We previously demonstrated that the median lethal dose at 30 days (LD_50/30_) with 95% confidence limits for female C57BL/6J mice was 7.52 Gy (7.44 Gy, 7.59 Gy) in the AFRRI ^60^Co facility [25]. Mice exposed to this level of total body irradiation display a rapid decline of all mature blood cell types, with a corresponding loss of hematopoietic progenitors in the bone marrow compartment [3,6]. Protein carbonylation is a key marker for protein oxidation, including in response to radiation exposure [15,26,27]. We therefore investigated protein carbonylation in the bone marrow at 24 h postirradiation (Figure 1). Two dimensional gel electrophoresis and Oxyblot analysis detected 13 carbonylated proteins in sham-irradiated and irradiated bone marrow samples. Twelve of these features were present in sham-irradiated bone marrow (spots 1–12) whereas feature 13 was only observed in the irradiated marrow sample. Interestingly, as compared to control, radiation exposure dramatically altered the pattern of protein carbonylation. The carbonylation status of 5 proteins was increased by radiation (spots 6, 9, 10, 12, and 13). Interestingly, the degree of carbonylation of 5 others was decreased following radiation exposure (spots 1, 2, 3, 4, and 5). The carbonylation levels of 3 proteins were relatively unchanged by radiation (spots 7, 8, 11). This indicates that radiation-induced carbonylation occurs in an order process, differentially effecting specific proteins in bone marrow. 

### 3.2. Identification of Carbonylated Proteins from Control and Irradiated Bone Marrow Tissue

Carbonylated proteins were excised from corresponding Coomassie-stained gels and subjected to peptide mass fingerprinting for protein identification. Definitive identifications were obtained only seven of the thirteen proteins, listed in Table 1. 

**Table 1:** Carbonylated proteins were identified by peptide mass finger printing. Numbers correspond to spot numbers identified in 2-D gels of control and irradiated bone marrow proteins (Figure 1). Symbols −, +, ++, +++ indicate relative levels of protein carbonylation in control *vs.* total body irradiation (7.5 Gy).

Spot No.	Protein ID	# Peptides	% Coverage	Control	Radiation
1	78 kDa glucose-regulated protein (GRP78)	22	37.5	+	−
2	Heat shock cognate 71 kDa protein (HSC71)	19	38.9	++	−
3	Heat shock cognate 71 kDa protein (HSC71)	29	52.5	++	−
6	Chitinase-like protein 3 (CHI3L1 or YKL-40)	19	53.8	+	++
7	Actin, cytoplasmic	15	49.6	+	+
8	Actin, cytoplasmic	10	29.3	+	+
10	Carbonic anhydrase 2 (CAII)	12	67.7	+	+++

The proteins identified here function as either enzymes or in protein-protein interactions. Protein carbonylation proteomics by others have been performed in studies of chemical- and radiation-induced oxidative injury as well as various disease states associated with oxidative stress, such as diabetes mellitus, neurodegenerative diseases, inflammatory diseases, and cancer [12,28,29,30,31]. Our current findings correlate with other investigations where enzymes were demonstrated to compose a large number of the identified carbonylated proteins. 

Chitinase-3-like 1 (CHI3L1/YKL-40) is a glycoprotein upregulated in response to immune system activation and is a blood biomarker for inflammation [32,33]. The function of CHI3L1 is controversial. In some cases, CHI3L appears inhibit inflammation, including suppression of the expression and secretion of interleukins [33]. However, other studies have shown the binding of CHI3L1 to interleukin receptors to induce macrophage activation [34]. Interestingly, chitinase was also shown to be upregulated in patients with asthma and other chronic inflammatory and remodeling diseases [35], but the carbonylation of CHI3L1 has not been previously demonstrated.

Carbonic anhydrase, a regulator of acid-base homeostasis, like actin, was previously shown to be carbonylated in skeletal muscle following high levels of ROS production as well as in some animal models of muscle dysfunction and in patients with chronic obstructive pulmonary disease [36,37]. 

The 78 kDa glucose-regulated protein (GRP78) is an ER chaperone that has been suggested to protect cells against damage by ROS; upregulation of GRP78 is associated with ER stress in cultured cells and *in vivo* in the brain, liver, and heart [38,39,40,41]. The heat shock cognate 71 kDa protein (HSC71) is also a chaperone, also demonstrated to be upregulated in response to redox stress [42] and HSC71 was demonstrated to contain increased carbonyl levels in Alzheimer’s disease [43]. HSC71 contains a reversibly oxidized cysteine that is responsive to ROS levels inside cells, but function of oxidation of the protein and the site of carbonylation relative to the oxidize cysteine are not yet known [44]. Both GFP78 and HSC71 were demonstrated to be oxidized in response to arsenic trioxide treatment of HTLV-1-infected HeLa cells in culture [45]. 

Finally, actin, a ubiquitous cellular protein required for normal biological function in all cell types, was previously shown to be carbonylated primary astrocytes following 1,3-dinitrobenzene exposure and in HL-60 human leukemia cells following peroxide-induced oxidative stress [19,29]. A recent investigation of oxidation and reduction thiols in cellular proteins found that actins contain a redox-sensitive cysteine, but it the structural site of the carbonylation is not yet known [44].

## 4. Conclusions

The findings presented here show that ionizing radiation differentially affects the carbonylation state of specific bone marrow proteins. The mechanism by which radiation causes a decrease in carbonylation of some proteins, but not others, remains unknown. Although carbonylation is largely considered to be an irreversible event, requiring protein degradation for its elimination, some recent reports suggest that carbonylated proteins may undergo further modification, suggesting a specific function for carbonylation [21,46]. The pattern of response that we observe suggests that radiation-induced carbonylation occurs is an ordered process, differentially effecting specific proteins and sparring others. The distinctive pattern of protein carbonylation in bone marrow may constitute a biosignature for radiation injury and suggests a causative mechanism for radiation-induced disruptions in hematopoiesis. While the number of proteins identified here is too small to assemble biochemical pathways underlying bone marrow pathology, the findings constitute a first step towards a more comprehensive understanding of how radiation can affect the functions of bone marrow.
